# The Association of Freezer Storage Time With Vitamin K and Vitamin D Concentrations in Human Brain Tissue

**DOI:** 10.1093/geroni/igaa057.473

**Published:** 2020-12-16

**Authors:** Kyla Shea, Xueyan Fu, Gregory Dolinkowski, Bess Dawson-Hughes, Thomas Holland, Klodian Dhana, Sarah Booth

**Affiliations:** 1 Tufts University, Boston, Massachusetts, United States; 2 Rush University, Chicago, Illinois, United States

## Abstract

Vitamins K and D are present in the human brain and have been implicated in Alzheimer’s disease and related dementias (ADRD). Because the use of banked brain tissue in ADRD research is increasing, we evaluated the stability of vitamin K and vitamin D in human brain tissue over long-term freezer storage using samples obtained from the Rush Memory and Aging Project (n=500, mean age=91, 29% male). Specimens were stored at -80□C until analyzed. Vitamin K (menaquinone-4, MK4) and vitamin D (25(OH)D) were measured in four regions (mid-temporal and mid-frontal cortexes, cerebellum, anterior watershed white matter) and averaged across regions. Storage time was categorized into two-year increments. Differences in MK4 and 25(OH)D concentrations according to storage time were evaluated using general linear models. MK4 concentrations did not differ in brains stored ≤8 years (geometric mean±SEM MK4 pmol/g: storage ≤2.0 years=1.2±0.1, 2.1-4.0 years=1.2±0.1, 4.1-6.0 years=1.4±0.1, 6.1-8.0 years=1.4±0.2; p≥0.21). MK4 in brains stored >8.0 years (0.8±0.1 pmol/g) was 33% lower than the concentration in brains stored ≤2.0 years (p=0.005). The 25(OH)D concentrations did not differ in brains stored ≤6 years (geometric mean±SEM 25(OH)D pmol/g: storage ≤2.0 years=1.2±0.1, 2.1-4.0 years=1.1±0.1, 4.1-6.0 years=1.2±0.1; p≥0.37). The 25(OH)D concentration in brains stored >6.0 years was 31-37% lower than that in brains stored ≤2.0 years (6.1-8.0 years=0.8±0.06, >8.0 years=0.7±0.04; p<0.001). MK4 and 25(OH)D appeared to be stable in human brain tissue stored at -80oC for up to 8 and 6 years, respectively. Storage time merits consideration when designing and interpreting studies that relate brain nutrient concentrations to ADRD.

